# Phenotypic and genetic analysis of milking temperament and its correlation with milk production traits in South African Holstein cattle

**DOI:** 10.1007/s11250-025-04437-0

**Published:** 2025-04-30

**Authors:** Thuli Tintswalo Siwele, Bohani Joseph Mtileni, Khathutshelo Agree Nephawe, Matome Andrias Madilindi, Bekezela Dube, Cuthbert Baldwin Banga

**Affiliations:** 1 Department of Animal Science, Tshwane University of Technology, Private Bag X680, Pretoria, 0001 South Africa; 2 Agricultural Research Council - Animal Production, Private Bag X2, Irene, 0062 South Africa; 3 Department of Animal Sciences, Faculty of Animal and Veterinary Sciences, Botswana University of Agriculture and Natural Resources, Private Bag 0027, Gaborone, Botswana; 4 Department of Agriculture and Animal Health, University of South Africa, Private Bag X6, Florida, 1710 South Africa

**Keywords:** Dairy cattle, Reactiveness, Genetic improvement, Milk composition, Welfare

## Abstract

Milking temperament (MT) is a crucial trait in dairy production; it affects farm profitability as well as animal and human welfare. Furthermore, poor temperament may increase herd costs by compromising the state and durability of the milking system. There is, however, limited knowledge and recording of MT in South African dairy cattle. The purpose of this study was to evaluate factors influencing MT scores and to estimate genetic parameters among MT and milk production traits (milk yield, fat and protein) in South African Holsteins. Data comprised of MT assessments on 2,844 cows from 16 herds collected between September 2020 and November 2021. Non-genetic effects were analysed by general linear models (GLM) procedure, and repeatability of MT scores was estimated using the variance components procedure of the Statistical Analysis Software. Linear animal models were fitted to estimate genetic parameters, using the ASReml software. Herd-test-day and age of cow at calving (*p* < 0.0001), and lactation stage (*p* < 0.05), significantly influenced MT. Repeatability estimate was moderate (0.47 ± 0.03), and the heritability estimate was low (0.05 ± 0.04). Heritability estimates were low to moderate for milk yield and composition, varying from 0.11 ± 0.05 for milk yield to 0.24 ± 0.06 for protein percent (%). Genetic correlation for MT with milk yield was moderate (0.60 ± 0.35). Low correlations were observed for MT with fat % (-0.12 ± 0.24) and protein % (-0.30 ± 0.32). There was no discernible genetic trend for MT in animals born from 2009 to 2019, although there was a minimal overall decline over the period. These findings suggest that there was reasonable consistency in the assessment of MT, and that MT may be improved through selection, using multi-trait models including milk yield.

## Introduction

Milking temperament (MT), or the type and degree of reaction of a cow to the milking procedure, is an increasingly important workability trait worldwide. It reflects level of comfort or stress experienced by the cow during milking, as indicated by its behavioural reaction and, therefore, is related to welfare (Wenzel et al. 2003; Szentleleki et al. 2015). Animals with favourable (calm) temperaments are easier to handle, feed, milk and transport (Santos et al. 2018; Costilla et al. 2020; Jaskowski et al. 2023). Extreme reactiveness (i.e., poor temperament) can endanger other animals and handlers; hence, temperament influences both animal and human welfare (Mincu et al. 2021; Smolinger and Skorjanc 2021). Besides its importance as a welfare-related trait, MT has large implications on herd profitability as it is associated with cow performance indicators such as milk yield and composition (Cziszter et al. 2016), and health (Santos et al. 2018). Furthermore, it is associated with survivability (Chang et al. 2019), reproduction (Sewalem et al. 2011; Cziszter et al. 2016), milking speed (Jacobsen et al. 2009; Kramer et al. 2013; Agravat et al. 2023) and performance in automated milking systems (Wethal and Heringstad 2019). Thus, there is a need for genetic improvement of MT in cattle populations.

Dairy breeding objectives worldwide are increasingly being broadened to incorporate functional and welfare-related traits that received little attention in the past (Miglior et al. 2017). Regarding the traits related to reproduction and health, there is a growing interest in management or workability traits such as MT due to their economic importance and association with welfare and ease of management (Szymik et al. 2015; Costilla et al. 2020). Milking temperament is now included in many dairy breeding programmes worldwide (e.g., United Kingdom, Denmark, Sweden, France and Norway) (Byrne et al. 2016; Chang et al. 2020; INTERBULL 2022). In South Africa, there is limited research on MT and no efforts have been made to include the trait in the national recording and genetic improvement programme (INTERBULL 2022).

Milking temperament has been found to exhibit genetic variation, which implies scope for genetic improvement through selection (e.g., Stephansen et al. 2018; Chang and Wang 2020; Antanaitis et al. 2021; Batista-Taborda et al*.* 2023). Heritability estimates for MT in the literature range from low to moderate, with most of them falling between 0.03 and 0.36 (Kramer et al. 2013; Stephansen et al. 2018; Chang and Wang 2020; Antanaitis et al. 2021; Batista-Taborda et al*.* 2023). Estimates based on subjective scores are generally low (0.03 to 0.14) (Cue et al. 1996; Sewalem et al. 2011; Rinell et al. 2014; Chang and Wang 2020; Antanaitis et al. 2021; Batista-Taborda et al*.* 2023) compared to those from automated machine connection data (0.26 to 0.36) (Stephansen et al. 2018). Scoring scale and analytical models applied also appear to contribute towards to the variability in estimates.

Accurate selection for MT may be achieved by multiple trait analysis with traits such as milk production, providing a reasonable genetic correlation. Genetic correlation estimates between MT and milk production traits are relatively few in the literature and range from low to moderate (Kruszynski et al. 2013; Chang et al. 2020; Antanaitis et al. 2021). Chang et al. (2020) reported a moderate range of genetic correlations, from 0.27 to 0.42, for MT with milk yield, protein and fat %, in Chinese Holsteins. Low estimates ranging from 0.01 to 0.07 were noted for Holsteins in Lithuania (Antanaitis et al. 2021) and Poland (Kruszynski et al. 2013). Most of the genetic correlations were positive, suggesting that selection for good MT (calm animals) might result in a correlated improvement in milk yield, protein and fat %, and vice versa.

The main aim of the current study was to estimate genetic parameters among MT and milk production traits in the South African Holstein cattle population. Holstein is the most widely used dairy cattle breed in South Africa, making up more than 60 per cent of the dairy cattle population (Banga et al. 2014). The estimates obtained in this study are a key prerequisite for including MT in the selection objective for South African Holstein cattle.

## Materials and methods

### Study population

Milking temperament was assessed on milking cows from 16 Holstein herds that are routinely recorded under the National Milk Recording and Improvement Scheme, from September 2020 to November 2021. These herds had similar milking systems and were a sample of commercial dairy farms in the South African provinces of Free State, Eastern Cape, Gauteng and KwaZulu-Natal. Cows in four of the herds were scored 3 or 4 times at monthly intervals, and the rest were assessed only once. Milk production and pedigree data of these cows were extracted from the Integrated Registration and Genetic Information System (http://www.intergis.agric.za/) of South Africa.

### Measurement of milking temperament

Milking temperament of each cow was scored on a five-point-scale, adopting the widely used and accepted method described by Gergovska et al. (2012), as explained in Table 1. All cows were assessed by one person during the afternoon milking. Scoring was done by observing the cows during milking, and due care was taken not to interfere with the milking process. Behaviour of the cow was assessed from when it entered the milking parlour and the udder was prepared for milking until the clusters were removed.

**Table 1:** Description of milking temperament scoring system (Gergovska et al. 2012)

Score	Definition	Description of the behavior
1	Very nervous	Very restless during milking process with kicking and lifting their legs
2	Nervous	The animal startled when humans approach
3	Medium	Cows were calm but they move a lot
4	Calm	Stand calm on the bedding, slash the tail
5	Very calm	Never showed restlessness, fully calm and obedient

### Data editing and preparation

A total of 3,850 MT records were collected from 2,844 cows in the first to third lactation. These cows had 35,379 corresponding test-day records of milk production traits. Records of milk yield of < 3.0 kg or > 50.4 kg, fat % of < 2.66% or > 5.56% and protein % of < 2.33% or > 4.44% were deleted from the dataset. Test-day records within 10–305 days in milk (DIM) were incorporated into the analyses (O'Callaghan et al. 2021; Madilindi et al*. *2023). Age of cow at calving (ACC) for 1st, 2nd and 3rd parity, ranged from 18 to 38, 39 to 58 and 59 to 78 months, respectively (Mostert et al. 2006; Dube et al. 2008). Furthermore, ACC was grouped into 6 classes [ACC1 = 18–28 months (mo), ACC2 = 29–38 mo, ACC3 = 39–48 mo, ACC4 = 49–58 mo, ACC5 = 59–68 mo and ACC6 = 69–72 mo]. Lactation stage (LS) was grouped into early (10–100 DIM), mid (101–200 DIM) and late (201–305 DIM). Herd and test date were concatenated to create herd-test-day (HTD), and considered as a contemporary group. Two separate data sets were created. Data set 1 comprised of 1,348 MT records of 363 cows from four herds, with each cow having 3 or 4 repeated scores. Data set 2 comprised of MT and test-day milk yield and composition records of 2,278 cows, with each cow having a single MT score. Data set 1 was used to compute the repeatability of MT scores and data set 2 was used for the remainder of the analyses. The pedigree file was built around animals in data set 2 with MT, milk yield, fat and protein % records, going three generations back. Contemporary groups (i.e. HTD) with less than 3 sires and 5 records were excluded. The final pedigree data set recorded 2,278 cows from 16 herds sired by 264 sires, and daughters of 1,309 dams.

### Data analysis

Summary statistics for MT, milk yield, fat and protein percent were calculated by the Proc Means procedure of the Statistical Analysis System (SAS) 9.4 (SAS, Institute, Carry, NC, USA). Repeatability of MT scores was calculated from data set 1, by the Varcomp Procedure of SAS 9.4, using the following equation (Caroli 1998):

1 $$Repeatability =\frac{\text{Var}(\text{Animal})}{\text{Var}\left(\text{Animal}\right)+\text{Var}(\text{Error})}=\frac{{\upsigma }_{\text{ANIMAL}}^{2}}{{\upsigma }_{\text{ANIMAL}}^{2}+{\upsigma }_{\text{ERROR}}^{2}}$$

where Var(Animal) is the variance of MT scores within animal; Var(Error) is the residual error variance.

Analysis of variance was conducted on data set 2, using the General Linear Models procedure of SAS 9.4 to establish non-genetic factors influencing MT. The effects tested were HTD, ACC, LS and parity, and the following model was applied:

2 $${y}_{ijklm}=\mu +{HTD}_{i}+ {LS}_{j}+ {P}_{k}+ {ACC}_{l}+ {e}_{ijklm}$$

where ${y}_{ijklm}$ is an observation for MT; µ is the underlying population mean;${HTD}_{i}$ is the fixed effect of contemporary group i; ${LS}_{j}$ is the fixed effect of LS i (i = early, mid, late); ${P}_{k}$ is the fixed effect of parity k (k = 1, 2, 3); ${ACC}_{l}$ is fixed effect of ACC group l [1 = ACC1 (18–28 mo), ACC2(29–38 mo), ACC3(39–48 mo), ACC4(49–58 mo), ACC5(59–68 mo) and ACC6(69–72 mo)]; e_ijklm_ is the residual error. Residual errors were assumed to be independent and identical, and distributed normally with mean of 0 and variance ${\sigma }_{e}^{2}$, i.e.:$e{}_{ \sim }{}^{iid}N(0,{\varvec{I}}{\sigma }_{e}^{2}$), where *σ*^*2*^_*e*_ is the residual variance and ${\varvec{I}}$ is an identity matrix. Significant means (*p* < 0.05) were separated using the least significant difference procedure (α = 0.05).

(Co)variance components among MT, milk yield, protein and fat % were estimated from data set 2, by linear animal models, using the Restricted Maximum Likelihood (REML) procedure of ASReml 4.2 (Gilmour et al. 2021). Single trait models were run first, to derive starting values, followed by bivariate analyses to estimate (co)variance components and genetic and phenotypic correlations for MT with milk yield, fat and protein %. The following general models, in matrix notation, were fitted:

3 $$\left[\begin{array}{c}{y}_{1}\\ {y}_{2}\end{array}\right]\text{=}\left[\begin{array}{cc}{X}_{1}& 0\\ 0& {X}_{2}\end{array}\right]\left[\begin{array}{c}{b}_{1}\\ {b}_{2}\end{array}\right]\text{+}\left[\begin{array}{cc}{Z}_{1}& 0\\ 0& {Z}_{2}\end{array}\right]\left[\begin{array}{c}{u}_{1}\\ {u}_{2}\end{array}\right]\text{+}\left[\begin{array}{c}{e}_{1}\\ {e}_{2}\end{array}\right],$$

where ${y}_{1}$ is a vector of phenotypic values for MT and ${y}_{2}$ is a vector of phenotypic value for milk yield, fat or protein %;${b}_{1}$ and ${b}_{2}$ are vectors of fixed effects affecting the traits; ${u}_{1}$ and ${u}_{2}$ are vectors of animal of genetic effects; ${e}_{1}$ and ${e}_{2}$ are vectors of random residual effects; ${X}_{1}$ and ${X}_{2}$ are incidence matrices for ${b}_{1}$ and ${b}_{2}$, respectively; ${Z}_{1}$ and ${Z}_{2}$ are incidence matrices for ${u}_{1}$ and ${u}_{2}$, respectively. Fixed effects of milk yield, fat and protein % were HTD, ACC, LS and parity (Mostert et al. 2006; Kgole 2013). The (co) variance matrix for random effects was defined as follows:

4 $$var\left[\begin{array}{c}u\\ e\end{array}\right]\text{=}\left[\begin{array}{cc}A{\sigma }_{u}^{2}& 0\\ 0& I{\sigma }_{e}^{2}\end{array}\right]$$

where the distribution of u was assumed to be *u* ~ *N* (0,$A{\sigma }_{u}^{2}$); *A* is the genetic additive correlation matrix; and ${\sigma }_{u}^{2}$ is the animal additive genetic variance. Residual effects (e) were assumed to be distributed with *N* ~ (0,${I\sigma }_{e}^{2}$), *I* is an identity matrix; and ${\sigma }_{e}^{2}$ is the residual variance and *COV* (*u, e*) = *0.*

Estimated breeding values (EBVs) for MT were computed for all animals in the pedigree file, from the co(variance) component estimates, by the Best Linear Unbiased Prediction method (Henderson 1984), using the ASReml 4.2 program (Gilmour et al. 2021). The genetic trend was then determined by plotting average EBVs by year of birth.

## Results

### Descriptive statistics

Summary statistics for MT, milk yield, fat and protein % are presented in Table 2. Milking temperament had a mean score of 3.05 ± 1.32, and the means for daily milk yield, fat and protein % were 27.22 ± 9.45, 3.84 ± 0.52 and 3.30 ± 0.34, respectively. Coefficient of variation was highest for MT (43.43%), followed by milk yield (34.73%). Protein % had the lowest coefficient of variation (10.39%). The repeatability estimate for MT scores was 0.47 ± 0.03.

**Table 2:** Descriptive statistics for milking temperament and milk production traits in South African Holsteins

Traits	Mean	SD	Minimum	Maximum	CV (%)	Repeatability
MT	3.05	1.32	1	5	43.43	0.47 ± 0.03
Milk (kg/day)	27.22	9.45	3	50.4	34.73	
Fat (%)	3.84	0.52	2.66	5.56	13.63	
Protein (%)	3.3	0.34	2.33	4.44	10.39	

*SD* standard deviation, *CV* coefficient of variation

### Environmental factors influencing milking temperament

**Fig. 1:**
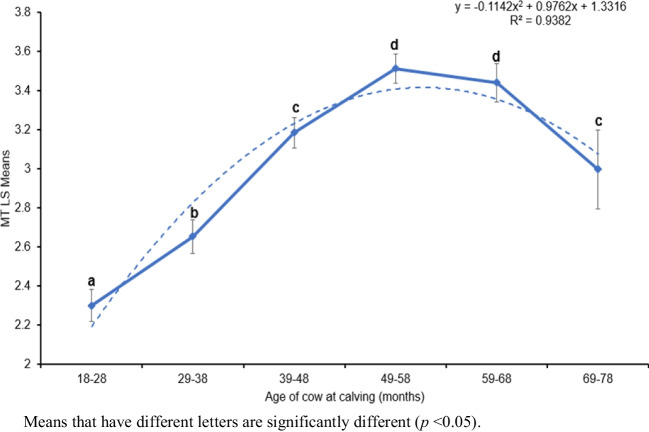
Least squares means for milking temperament by age of cow at calving

**Fig. 2:**
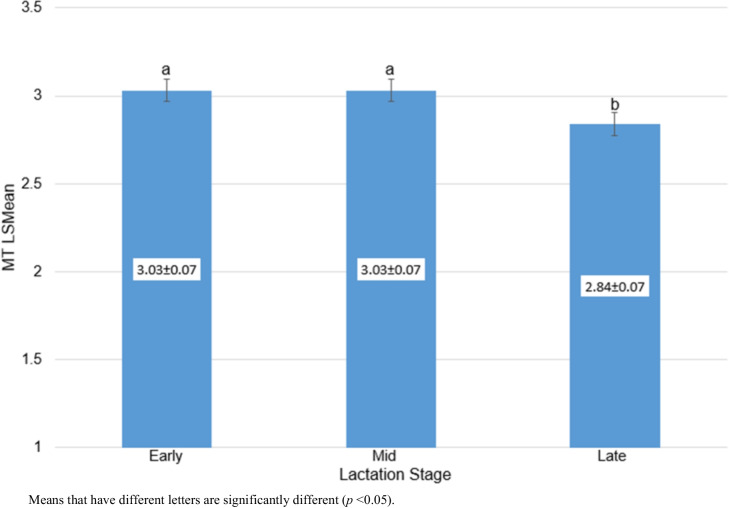
Least squares means for milking temperament by lactation stage

Table 3 outlines the effects of the environmental factors influencing MT. Herd-test-day was significant (*p* < 0.0001) and contributed 42% of the total variation in MT. Mean scores for HTD groups ranged from 1.13 ± 0.86 to 4.61 ± 1.22. Age of cow at calving also significantly influenced (*p* < 0.0001) MT. Figure 1 shows the trend of the least squares means for MT with ACC. Milking temperament scores increased with age, peaking at 49–50 months of age, then started to decline with advancing age. Lactation stage showed a significant influence (*p* < 0.05) on MT. Least squares means for MT scores by LS are illustrated in Fig. 2. Scores were significantly (*p* < 0.05) higher in early and mid compared to late lactation. However, no significant differences (*p* > 0.05) were shown between early and mid-lactation for MT. Animals in early and mid-lactation stages had a mean score of 3.03 (medium), while animals in late lactation tended to be slightly more nervous (mean = 2.84).

**Table 3:** Environmental factors influencing milking temperament in South African Holsteins

Factor	Mean Squares	*F* Value	*P*-value
HTD	6.54	4.41	< 0.0001
ACC	358.85	241.97	< 0.0001
LS	6.88	4.64	< 0.05
Parity	8.79	4.40	> 0.05

*HTD* herd-test-day, *LS* Lactation stage, *ACC* Age of cow at calving

Means that have different letters are significantly different (*p* < 0.05)

Means that have different letters are significantly different (*p* < 0.05).

### Genetic parameter estimates

#### Heritability

Table 4 presents the heritability estimates for MT and milk production traits. Milking temperament had a low heritability estimate of 0.05 ± 0.04, whereas those for milk yield, fat and protein % were low to moderate, varying from 0.11 ± 0.05 for milk yield to 0.24 ± 0.06 for protein %.

**Table 4:** Heritability estimates for milking temperament, milk yield, fat and protein percent in South African Holsteins

Trait	h^2^ ± SE
MT	0.05 ± 0.04
Milk (kg/day)	0.11 ± 0.05
Fat (%)	0.13 ± 0.05
Protein (%)	0.24 ± 0.06

*MT* milking temperament, *h*^*2*^ heritability, *SE* standard error

#### Genetic and phenotypic correlations

Estimated genetic and phenotypic correlations between MT and milk production traits are presented in Table 5. Genetic correlation was moderate and positive for MT and milk yield (0.60 ± 0.35). Negative genetic correlations were obtained between MT and fat (− 0.12 ± 0.24) and protein % (− 0.30 ± 0.32). The corresponding phenotypic correlations were much lower (− 0.04 ± 0.02 to 0.25 ± 0.02), although the direction of the relationships remained similar.

**Table 5:** Genetic and phenotypic correlations between milking temperament and milk yield, fat and protein percent in South African Holsteins

Trait	r_g_ ± SE	r_p_ ± SE
Milk (kg/day)	0.60 ± 0.35	0.25 ± 0.02
Fat (%)	− 0.12 ± 0.24	− 0.06 ± 0.03
Protein (%)	− 0.30 ± 0.32	− 0.04 ± 0.02

r_g_ = Genetic correlation; r_p_ = Phenotypic correlation; SE = Standard error

#### Genetic trend

**Fig. 3:**
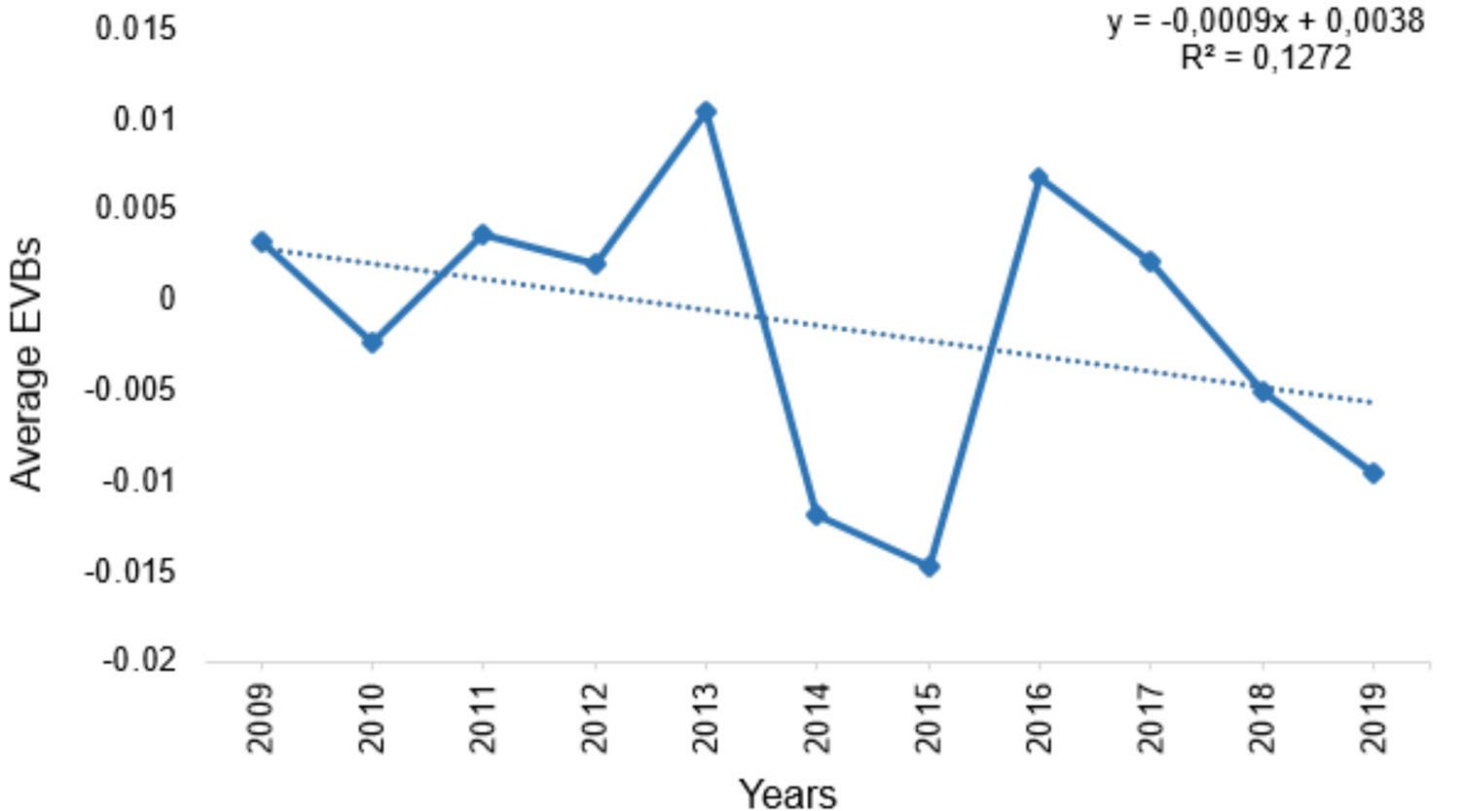
Genetic trend for milking temperament in South African Holstein cattle

Figure 3 shows the genetic trend for MT, estimated from cows born from 2009 to 2019. There was no consistent trend, with average EBVs fluctuating over the period. In 2013, there was a noticeable peak in average EBVs, and a deep decline was observed in 2014–2015. Overall, there was a decrease in mean EBV, at the rate of 0.0009 each year, in the 10-year period. The coefficient of determination for the linear best fit equation was, however, very low (R^2^ = 12.72%).

## Discussion

### Descriptive statistics

Milking temperament is an important functional trait in dairy cattle because it influences animal and human welfare as well as herd profitability. There is also evidence that it may be associated with milk production traits. In the current work, cows were evaluated for MT with scores from 1 “very aggressive/nervous” to 5 “very calm”. Milking temperament had a mean score of 3.05 ± 1.32, meaning that, on average, cows in the study were moderately calm. An earlier study observed very calm cows (4.54 ± 0.63) in Hungarian Holstein Friesian (Szentleleki et al. 2015). Scoring systems dissimilar to the one employed in the current study have, however, been used in most of the previous studies (e.g. Kruszynski et al. 2013; Dutt et al. 2016; Wethal and Heringstad 2019; Antanaitis et al. 2021; Szymik et al. 2021; Batista-Taborda et al. 2023), which makes it difficult to compare mean scores.

Coefficient of variation for MT (43.43%) was much higher than for production traits, indicating comparatively large variability in MT scores. There were, however, no estimates of coefficient of variation for MT available in the literature to compare with. Milk production traits had estimates of coefficient of variation ranging from 10.39% for protein to 34.73% for milk yield, compared to values obtained recently by Ismael (2021) in Serbian Holstein–Friesian cows.

### Repeatability estimate for milking temperament scores

Repeatability of MT was estimated mainly to assess how closely successive scores on the same cow agree. This depends mostly on the consistency of the person doing the assessment, although temporary environmental effects on the animal may also have some influence. In the current study, all the cows were assessed by one person. Martin and Bateson (1986) proffered that repeatability estimates from 0.2 to 0.4 are low, between 0.4 and 0.7 are moderate; and larger than 0.7 are high. A moderate repeatability estimate (0.47 ± 0.03) was obtained for MT scores in this study, in close agreement with other studies elsewhere (Kramer et al. 2013; Wethal and Heringstad 2019; Polupan et al. 2021; Batista-Taborda et al*.* 2023). The moderate repeatability estimate suggests that repeated scores of MT on a cow are fairly consistent. In addition to indicating consistence of the assessor, this means that it may not be necessary to assess MT repeatedly on a cow, since the first score corresponds reasonably with future scores.

### Non-genetic factors influencing milking temperament

Non-genetic factors affecting MT were determined mainly to fit them in predicting genetically. The factors identified were HTD, ACC, and LS, and these were subsequently included in the models estimating genetic parameters for MT.

Herd-test-day turned out to be the most significant sources of variation in MT, accounting for 42% of the total variation, which is in agreement with several other studies (Rensing and Ruten 2005; Szentleleki et al. 2008; Sewalem et al. 2011; Haskell et al. 2014; Wethal and Heringstad 2019; Antanaitis et al. 2021). This effect may be attributable to management factors such as animal handling, milking procedure and human approach, which may differ among herds and within the same herd on a day-to-day basis (Constatini 2008; Antanaitis et al. 2021; Jaskowski et al. 2023).

Age of the cow at calving came out as another factor with a major influence on MT, in concurrence with several previous studies (Turner et al. 2013; Neja et al. 2015; Caetano et al. 2017; Cielava et al. 2017; Eastham et al. 2018; Almasri et al. 2020). In the current study, MT scores increased (i.e. cows became calmer) with increase in age of the cow, reaching a peak at 49–68 months of age, and then started to decline (i.e. cows became less calm) with advancing age. This trend could be because young cows are naturally more nervous, and less familiar with the overall milking procedure than older cows (Rousing et al. 2004; Haskell et al. 2012; Marcal-Pedroza et al. 2023) and thus require more management focus. However, contrary to the current study, it was reported that older Polish Holsteins were more aggressive during the milking process compared to younger cows (Kalinska and Slosarz 2016; Karamfilov 2022). The reason for this might be that older cows may become uncomfortable due to swollen udders from the pressure of increased level of daily milk yield (Szentleleki et al. 2015).

Milking temperament was also significantly influenced by LS, with cows in early and mid-lactation being calmer than those in late lactation. This is consistent with previous findings by Neja et al. (2015) in Polish Holstein–Friesian cattle, and may be due to the drop in milk production in late lactation causing cows to be uncomfortable and stressed during the milking process (Chebel et al. 2016; Proudfoot et al. 2018). Some researchers have, however, observed relatively more nervousness/aggressiveness in early-lactation cows than those in late-lactation (Gergovska et al. 2012; Sawa et al. 2017; Antanaitis et al. 2021). This could be partly caused by the stress of adjusting to new groups, and increased physiological stress, during early lactation.

### Heritability estimates for milking temperament scores and milk production traits

A key objective of this work was to determine the extent to which MT is under additive genetic effects, so as to establish the potential for genetic improvement through selection. The (co)variance component estimates derived during the analysis also provide the basis for estimating breeding values for MT in the South African Holstein population. Estimated heritability for MT was low (0.05 ± 0.04), in agreement with several other studies which reported estimates varying from 0.03 to 0.14 (Cue et al. 1996; Sewalem et al. 2011; Rinell et al. 2014; Chang and Wang 2020; Antanaitis et al. 2021; Batista-Taborda et al*.* 2023). These studies were also based on subjectively assessed MT, and factors such as the scoring scale and analytical models applied appear to account for the slight variation in these estimates. Larger estimates of heritability, ranging between 0.26 and 0.36 were reported for first-parity Danish Holstein cows, based on MT data from objective automatic machine connection evaluation (Stephansen et al. 2018). It is, however, difficult to directly compare these estimates with those obtained in the current and other previous studies, due to the disparity in the scoring methods used. Nevertheless, the higher estimates observed by Stephansen et al. (2018) may suggest that objective assessment of MT is better at capturing genetic variance among individual animals, or reducing error variance. This assessment procedure is, however, more time-consuming and can only be used for herds with automatic milking machines. It is evident from the current study and the literature that MT is under some genetic influence. The low heritability estimate obtained for MT in South African Holstein cows may imply low accurate selection. This can, however, be improved through approaches such as multi-trait analysis including correlated traits. For instance, Eaglen et al. (2012) showed that multiple trait analyses including gestation period, calving ease and stillbirth had higher prediction accuracy than univariate analysis for calving ease and stillbirth.

Heritability estimates for milk production traits in the current study ranged from low (0.11 ± 0.05) for milk yield to moderate (0.24 ± 0.06) for protein %, and were comparable to those from earlier studies in South African Holsteins (Makgahlela et al. 2007; Maiwashe et al. 2008; Kgole 2013; Tlabela 2020; Van Niekerk et al. 2023) and elsewhere (Peixoto et al. 2016; Getahun et al. 2020; Ismael et al. 2021; Batista-Taborda et al*.* 2023; Kinghorn et al. 2023). The relatively higher heritability of these traits may render them useful for improving selection accuracy for MT using multi-trait analysis, provided there is considerable genetic correlation.

### Genetic and phenotypic correlations for milking temperament scores with milk production traits

Strong genetic correlations between MT and milk production traits may provide means to improve the accuracy of selection for MT through multiple trait analysis. In the current study, MT had a moderate positive relation with milk yield, suggesting that cows with good temperament tended to produce more milk, and vice versa, which is in concurrence with other previous studies (Sawa et al. 2017; Agravat et al. 2023). Comparable findings were reported for Chinese Holsteins by Chang et al. (2020), and imply that selection for increased milk yield will probably result in a correlated improvement in MT. Furthermore, accuracy of selection for MT may be improved through multi-trait analysis including milk yield. Kruszynski et al. (2013) also found a positive genetic interrelation between MT and milk yield in Brown Swiss cattle, although the relationship was very weak. Contrary to all these studies, Antanaitis et al. (2021) observed a negative, although very low, genetic association between MT and milk yield in Lithuanian Holsteins.

Genetic correlations among MT and fat and protein % were low to moderate, and both negative. The unfavourable relationship is plausible, given the fact that fat and protein % are negatively correlated with milk yield and MT had a positive association with milk yield. Some earlier studies elsewhere have, however, reported favourable relationships between MT and fat and protein % (Kruszynski et al. 2013; Sawa et al. 2017; Chang et al. 2020; Antanaitis et al. 2021; Agravat et al. 2023).

Phenotypic correlations between MT and milk production traits followed the same trend as the genetic correlations, but were much lower. The estimate for milk yield was much higher, whereas those for fat and protein % were comparable, relative to those from a previous study on Lithuanian Holsteins (Antanaitis et al. 2021). It, therefore, appears that the relationship between MT and fat and protein % is weak at both the genetic and phenotypic levels.

### Genetic trend for milking temperament

Genetic trend for MT was estimated to ascertain whether there has been any changes in genetic value for MT in the South African Holstein population in recent years. No consistent trend was observed and, overall, there was a slight genetic merit decrease for MT during the 10 year duration 2009–2019. Consistent increases in the genetic trend for milk yield have been reported in the South African Holstein population (Ramatsoma et al. 2014). It was therefore expected that the genetic trend for MT would also increase, given the positive genetic correlation between milk yield and MT in the current study. The slight decline observed could be due to the fact that there was no selection for MT in the South African Holstein population. Similar results were also reported in Polish Holstein–Friesian (Kruszynski et al. 2013); however, a minor albeit also inconsistent increase was observed in Canadian Holstein (Sewalem et al. 2011). It is, therefore, imperative to include MT in the breeding programme for South African Holstein cattle, so as to achieve meaningful genetic improvement.

## Conclusion

Herd-test-day, age of cow at calving, and lactation stage have major influences on MT and, therefore, ought to be accounted for in genetic evaluation models. The subjective scoring of MT used in the current study showed reasonable consistency and, therefore, can be applied as a reliable phenotyping procedure. The observed low heritability estimate for MT suggests that, through selection, genetic progress may be slowly achieved. However, the moderately high genetic correlation between MT and milk yield may be an opportunity to improve selection accuracy for MT by multi-trait analysis including both traits. Implementation of large-scale recording of MT on South African Holstein herds is required, as another approach to improve the accuracy of selection. No distinct change in the genetic value for MT was observed in the Holstein population in South Africa, in recent years, highlighting the need to include the trait in the selection objective.

## Data Availability

The datasets generated during and/or analysed during the current study are available from corresponding author on reasonable request.
